# New Insight into Phase Formation of M_*x*_Mg_2_Al_4+*x*_Si_5−*x*_O_18_:Eu^2+^ Solid Solution Phosphors and Its Luminescence Properties

**DOI:** 10.1038/srep12149

**Published:** 2015-07-20

**Authors:** Jun Zhou, Zhiguo Xia, Mingyue Chen, Maxim S. Molokeev, Quanlin Liu

**Affiliations:** 1 School of Materials Sciences and Technology, China University of Geosciences, Beijing 100083, China; 2 School of Materials Sciences and Engineering, University of Science and Technology Beijing, Beijing 100083, China; 3 Laboratory of Crystal Physics, Kirensky Institute of Physics, SB RAS, Krasnoyarsk 660036, Russia; 4 Department of Physics, Far Eastern State Transport University, Khabarovsk, 680021 Russia

## Abstract

Here we reported the phase formation of M_*x*_Mg_2_Al_4+*x*_Si_5−*x*_O_18_:Eu^2+^ (M = K, Rb) solid solution phosphors, where M^+^ ions were introduced into the void channels of Mg_2_Al_4_Si_5_O_18_ via Al^3+^/Si^4+^ substitution to keep the charge balance. XRD results revealed that the as-prepared phosphors with different M^+^ contents were iso-structural with Mg_2_Al_4_Si_5_O_18_ phase. The combined analysis of the Rietveld refinement and high resolution transmission electron microscopy (HRTEM) results proved that M^+^ ions were surely introduced into the intrinsic channels in Mg_2_Al_4_Si_5_O_18_. The emission peaks of M_*x*_Mg_2_Al_4+*x*_Si_5−*x*_O_18_:Eu^2+^ (M = K, Rb) phosphors with various *x* values performed a systematic red-shift tendency, which was ascribed to the elongation of [MgO_6_] octahedra. The temperature stable photoluminescence and internal quantum efficiency (QE) of M_*x*_Mg_2_Al_4+*x*_Si_5−*x*_O_18_:Eu^2+^ (M = K, Rb) phosphors were enhanced owing to the filling of M^+^ in the void channels suggesting a new insight to design the solid solution phosphors with improved photoluminescence properties.

Silicates have attracted great attention in recent years due to their high chemical stability, heat stability, low cost, excellent weather resistance and variety of crystal structures^1^. Amongst them, cordierite silicates have been widely used as a high quality refractory materials, integrated circuit board, catalyst carrier, ceramic foam and aviation materials etc., which are attributed to its easy preparation, fire resistance, good thermal shock resistance, and mechanical properties resistant to corrosion at higher temperature^2^,^3^. The cordierite compound is represented by a magnesium/aluminum aluminosilicate with the crystallo-chemical formula Mg_2_^[6]^Al_3_^[4]^(Si_5_Al^[4]^O_18_). Herein, Mg_2_Al_4_Si_5_O_18_ has a complex structure with six tetrahedral units [(Si/Al)O_4_], forming Si_6_O_18_-type 6-membered rings with one Al substituted for one Si in the ring^4^,^5^,^6^,^7^,^8^,^9^. Binding of the tetrahedra ring ensured by the [MgO_6_] octahedra and [AlO_4_] tetrahedra. Up to now, several studies regarding the luminescence properties of rare earth doped Mg_2_Al_4_Si_5_O_18_ were reported in the literature. Thim *et al.* studied the luminescence properties of Eu^3+^ doped Mg_2_Al_4_Si_5_O_18_^4^ After that, Chen *et al.* reported the spectroscopic properties of Eu^2+^ and Mn^2+^ in Mg_2_Al_4_Si_5_O_18_ by UV excitation^5^,^6^, and Ci. *et al.* investigated the white-emitting Mg_2_Al_4_Si_5_O_18_:Dy^3+^ phosphors^7^. In addition, Lü *et al.* furthered studied the full-colour phosphors Mg_2_Al_4_Si_5_O_18_ and K_0.26_Mg_2_Al_4.26_Si_4.74_O_18_, resulting from the emission of red (Mn^2+^), green (Tb^3+^) and blue (Eu^2+^/Ce^3+^) light^8^,^9^.

It is worth mentioning that Mg_2_Al_4_Si_5_O_18_ and K_0.26_Mg_2_Al_4.26_Si_4.74_O_18_ possess the same crystal structure. However, to the best of our knowledge, the relationship between the phases of Mg_2_Al_4_Si_5_O_18_ and K_0.26_Mg_2_Al_4.26_Si_4.74_O_18_, and the effect of K^+^ addition on the phase transformation has not been reported. Herein, we have fabricated M_*x*_Mg_2_Al_4+*x*_Si_5−*x*_O_18_:Eu^2+^ (M = K, Rb) phosphors, and their detailed crystal structures were comparatively investigated. A correlation between the crystal structures and luminescence properties for two series of phosphors has been discussed, which can demonstrate the new insight into understanding the structure-property relationship of the rare earth luminescence materials. It is further believed that the compositionally-optimized M_*x*_Mg_2_Al_4+*x*_Si_5−*x*_O_18_:0.03Eu^2+^ (M = K, Rb) phosphor can be potentially applied as the blue-emitting component in white-emitting diodes (*w*-LEDs).

## Results

### Phase and crystal structure analysis

**Figure 1:**
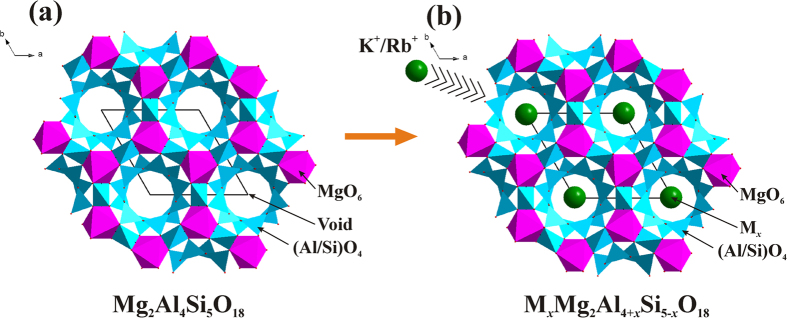
Schematic crystal structure diagrams of Mg_2_Al_4_Si_5_O_18_ with channel void along *c*-axis (**a**) and M_*x*_Mg_2_Al_4+*x*_Si_5−*x*_O_18_ compounds showing the existence of K^+^/Rb^+^ doping into the void (**b**).

Figure 1 presents the structural diagram of Mg_2_Al_4_Si_5_O_18_ and typical compound of M_*x*_Mg_2_Al_4+*x*_Si_5−*x*_O_18_, respectively. As can be seen from Fig. 1a, Mg_2_Al_4_Si_5_O_18_ belongs to hexagonal structure and has void channels along *c*-axis. In the structure, the [MgO_6_] octahedral was surrounded by the Si_6_O_18_-type 6-membered rings, which consists of corner-shared tetrahedra of [AlO_4_] and [(Si/Al)O_4_]. There is no direct bonding between rings, but they are linked by [MgO_6_] and by [AlO_4_] above and below. The Si atoms in the rings are bonded to two oxygen atoms in layers above and below and to two oxygen atoms in the ring. To the best of our knowledge, there are no further reports on this void channels and their potential optical application. Based on this, we tried to synthesize M_*x*_Mg_2_Al_4+*x*_Si_5−*x*_O_18_:Eu^2+^ (M = K, Rb) with different contents by introducing M^+^ into the void channels and using Al^3+^ and Si^4+^ to balance the charge.

**Figure 2:**
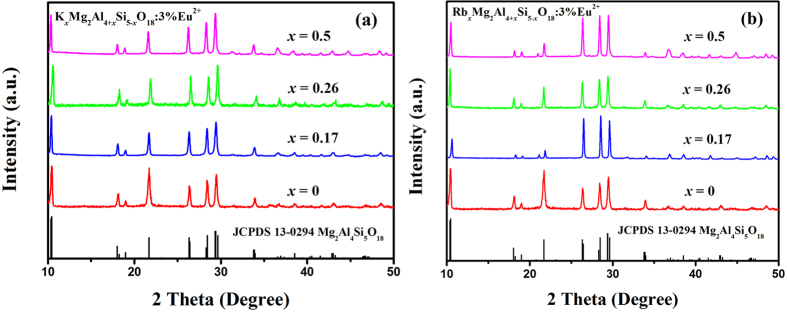
XRD patterns of as-prepared K_*x*_Mg_2_Al_4+*x*_Si_5−*x*_O_18_:0.03Eu^2+^ (*x* = 0, 0.17, 0.26 and 0.5) (**a**) and Rb_*x*_Mg_2_Al_4+*x*_Si_5−*x*_O_18_:0.03Eu^2+^ (*x* = 0, 0.17, 0.26 and 0.5) (**b**). The standard data for Mg_2_Al_4_Si_5_O_18_ (JCPDS card no. 13-0294) is also shown as a reference.

Figure 2a,b shows the typical XRD patterns of the as-prepared K_*x*_Mg_2_Al_4+*x*_Si_5−*x*_O_18_:0.03Eu^2+^ (*x* = 0, 0.17, 0.26 and 0.5) and Rb_*x*_Mg_2_Al_4+*x*_Si_5−*x*_O_18_:0.03Eu^2+^ (*x* = 0, 0.17, 0.26 and 0.5) samples, respectively. It can be found that all the diffraction peaks of these two series of samples can be exactly assigned to the corresponding standard data for hexagonal phase of Mg_2_Al_4_Si_5_O_18_ (JCPDS 13-0294), suggesting that doped M^+^ ions have been successfully dissolved in the Mg_2_Al_4_Si_5_O_18_ host lattice. However, once the occupation of the Eu^2+^ in the Mg_2_Al_4_Si_5_O_18_ host and M_*x*_Mg_2_Al_4+*x*_Si_5−*x*_O_18_ host is concerned, there are two different viewpoints, one is that the Eu^2+^ can enter the void channels, and the other viewpoint think that Eu^2+^ will replace the cations. Piriou *et al.* demonstrated that the Eu ion cannot enter the void channels based on the site-selective spectroscopy^10^. In the present case, it is assumed that Eu^2+^ (*r* = 1.17 Å when coordinate number (CN) = 6) ions will occupy the Mg^2+^ (*r* = 0. 72 Å when CN = 6) sites, because both the Al^3+^ (*r* = 0.39 Å when CN = 4) and Si^4+^ (*r* = 0.26 Å when CN = 4) sites are too small to accommodate the Eu^2+^ ions. In order to further analyze the crystal structure of the as-prepared samples, the Rietveld structural refinement for these samples were performed using TOPAS 4.2 (Bruker AXS TOPAS V4: General profile and structure analysis software for powder diffraction data. – User’s Manual. Bruker AXS, Karlsruhe, Germany. 2008.). Figure S1 (electronic supporting information) demonstrates the observed, calculated, and difference patterns. Based on the Rietveld refinement results, negligible amounts of impurity phases were identified in the samples, and all of these samples exhibit the same crystalline hexagonal crystal system with a space group *P*6*/mcc*. The final weighted *R* factors (*R*_wp_) of the samples were successfully converged at a satisfactory level, and the refined structural parameters of these samples are listed in Table S1. The unit cell parameters and Al^3+^/Si^4+^ ratio in tetrahedra become larger with increasing M^+^ content, which is ascribed to the fact that the M^+^ were introduced into the void channels. Furthermore, increasing Al^3+^ concentration results in the expansion of the [(Al/Si)O_4_] tetrahedra since the average bond length *d*(Al–O) = 1.618 Å is bigger than the average bond length *d*(Si–O) = 1.596 Å. In order to further understand the lattice mismatch between the Mg_2_Al_4_Si_5_O_18_ phase and the M_*x*_Mg_2_Al_4+*x*_Si_5−*x*_O_18_ (M = K, Rb) phase, Figure S2 gives the lattice variation of the M_*x*_Mg_2_Al_4+*x*_Si_5−*x*_O_18_ (M = K, Rb) phase compared to the original Mg_2_Al_4_Si_5_O_18_ phase. When M^+^ ions are introduced into the void channels of Mg_2_Al_4_Si_5_O_18_ via the synergistic Al^3+^/Si^4+^ substitution, the lattice parameter *a* for K_*x*_Mg_2_Al_4+*x*_Si_5−*x*_O_18_:Eu^2+^ and Rb_*x*_Mg_2_Al_4+*x*_Si_5−*x*_O_18_:Eu^2+^ series both increased, whereas the lattice parameter *c* for the two series of samples decreased. Such a different lattice variation will lead to the distortion of the corresponding polyhedra, which will be discussed later.

**Figure 3:**
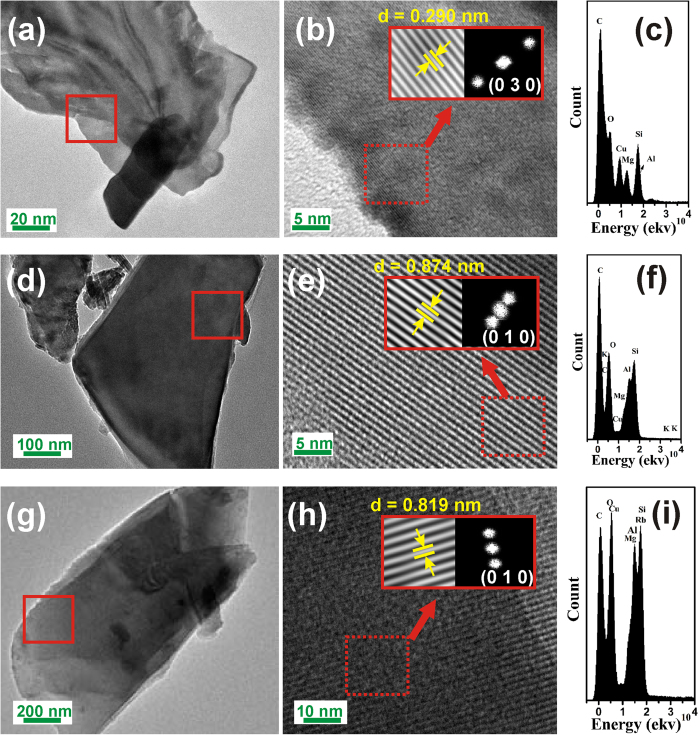
TEM images (**a**, **d** and **g**), the corresponding HRTEM images (**b**, **e** and **f**) and the EDS analysis on a single crystal (**c**, **f** and **i**) of typical Mg_2_Al_4_Si_5_O_18_:0.03Eu^2+^, K_0.17_Mg_2_Al_4.17_Si_4.83_O_18_:0.03Eu^2+^ and Rb_0.17_Mg_2_Al_4.17_Si_4.83_O_18_:0.03Eu^2+^ samples. The corresponding enlarged lattice fringes and their FFT patterns of the HRTEM images are given in the respective inset.

Coexistence and the element distribution of Mg, Al, Si, O, K and Rb in K_*x*_Mg_2_Al_4+*x*_Si_5−*x*_O_18_:0.03Eu^2+^ and Rb_*x*_Mg_2_Al_4+*x*_Si_5−*x*_O_18_:0.03Eu^2+^ series was further examined and verified by the HRTEM and the corresponding Energy Dispersive X-ray Spectrometry (EDS) analysis as shown in Fig. 3a,d,g show the TEM images obtained from the selected samples, Mg_2_Al_4_Si_5_O_18_:0.03Eu^2+^, K_0.17_Mg_2_Al_4.17_Si_4.83_O_18_:0.03Eu^2+^ and Rb_0.17_Mg_2_Al_4.17_Si_4.83_O_18_:0.03Eu^2+^. The fine structures of typical phosphor samples are further studied by HRTEM technique, and the fast Fourier transform (FFT) images suggest that the highly single-crystalline nature, as shown in Fig. 3b,e,h. It could be seen that the continuous lattice fringes measurements with *d* spacing of 0.290 nm, 0.874 nm and 0.819 nm agree well with the structure of the compound by refinement, and they could be assigned to the corresponding (0 3 0), (0 1 0) and (0 1 0) planes for Mg_2_Al_4_Si_5_O_18_:0.03Eu^2+^, K_0.17_Mg_2_Al_4.17_Si_4.83_O_18_:0.03Eu^2+^ and Rb_0.17_Mg_2_Al_4.17_Si_4.83_O_18_:0.03Eu^2+^, respectively. The EDS results (Fig. 3c,f,i) confirm the presence of all the elements (including the C and Cu from the sample holder), which were detected from one complete microcrystal (red square region) (Fig. 3c,f,i). The Eu element can’t be detected clearly due to its low concentration, but it can be confirmed by the emission spectra in the following section.

### Photoluminescence analysis

**Figure 4:**
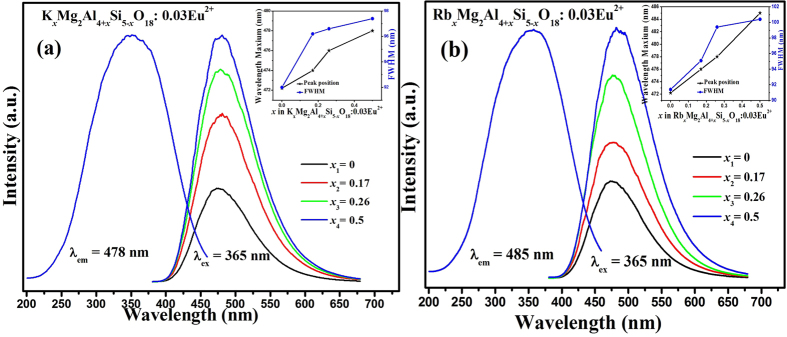
PLE and PL spectra of K_*x*_Mg_2_Al_4+*x*_Si_5−*x*_O_18_:0.03Eu^2+^ (**a**) and Rb_*x*_Mg_2_Al_4+*x*_Si_5−*x*_O_18_:0.03Eu^2+^ (**b**) phosphors; and the inset shows the corresponding M^+^ content dependent peak positions and FWHM values.

**Figure 5:**
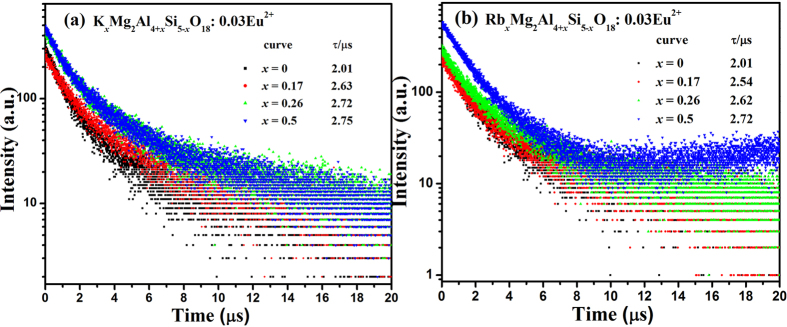
Room temperature decay curves of K_*x*_Mg_2_Al_4+*x*_Si_5−*x*_O_18_:0.03Eu^2+^ (**a**) and Rb_*x*_Mg_2_Al_4+*x*_Si_5−*x*_O_18_:0.03Eu^2+^ (**b**).

Room temperature photoluminescence excitation (PLE) spectra and photoluminescence emission (PL) spectra of the M_*x*_Mg_2_Al_4+*x*_Si_5−*x*_O_18_:0.03Eu^2+^ (*x* = 0−0.5) systems depending on M^+^-doping concentration are shown in Fig. 4a (M = K) and Fig. 4b (M = Rb), respectively. The PLE spectra of the M_*x*_Mg_2_Al_4+*x*_Si_5−*x*_O_18_:0.03Eu^2+^ (M = K, Rb) phosphors with optimal Eu^2+^ content monitored at their strongest emission wavelength of 478 and 485 nm show characteristic excitation bands of Eu^2+^ ion, which is broad in the range from 200 nm to 450 nm. Upon the excitation of 365 nm, the PL spectra of M_*x*_Mg_2_Al_4+*x*_Si_5−*x*_O_18_:0.03Eu^2+^ (M = K, Rb) exhibit blue emission bands, which are attributed to the 4f^6^5d-4f^7^ transition of the Eu^2+^ ion^11^. On the other hand, both the emission intensities and Full Width Half Maximum values (FWHM) were gradually enhanced as the M^+^ concentration increases from *x* = 0 to *x* = 0.5 as shown in the inset of Fig. 4, indicating that emission centers experienced a stronger crystal field strength^12^. At the same time, the emission bands of all the phosphors with various *x* values have systematic red-shift tendency from 472 nm to 478 nm for K_*x*_Mg_2_Al_4+*x*_Si_5−*x*_O_18_:0.03Eu^2+^, and from 472 nm to 485 nm for Rb_*x*_Mg_2_Al_4+*x*_Si_5−*x*_O_18_:0.03Eu^2+^, respectively, as given in the inset of Fig. 4a,b. Therefore Rb^+^ leads to bigger red-shift amount than that of K^+^, which could be due to the difference of the ion radii of Rb^+^and K^+^, so that the larger structural distortion can be expected. Furthermore, Fig. 5 presents the room temperature decay curves of Eu^2+^ luminescence in K_*x*_Mg_2_Al_4+*x*_Si_5−*x*_O_18_:0.03Eu^2+^ (a) and Rb_*x*_Mg_2_Al_4+*x*_Si_5−*x*_O_18_:0.03Eu^2+^ (b) series, respectively. All the decay curves can be well fitted with a second order exponential equation:


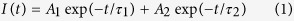


where *I* is the luminescence intensity, *A*_*1*_ and *A*_*2*_ are constants, *τ* is the time, *τ*_*1*_ and *τ*_*2*_ are rapid and slow lifetimes for exponential components, respectively. Furthermore, the effective lifetime constant (τ^*^) can be calculated as:


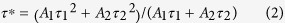


Based on the decay curves in Fig. 5 and fitting equation in Eq. (1) and the calculation equation in Eq. (2), the effective decay time (τ^*^) were determined to be 2.01, 2.63, 2.72 and 2.75 μs for K_*x*_Mg_2_Al_4+*x*_Si_5−*x*_O_18_:0.03Eu^2+^ with *x* = 0, 0.17, 026 and 0.5, respectively. And as for Rb_*x*_Mg_2_Al_4+*x*_Si_5−*x*_O_18_:0.03Eu^2+^, the lifetime values were 2.01, 2.54, 2.62 and 2.72 μs with *x* = 0, 0.17, 026 and 0.5, respectively. It was found that the lifetime values didn’t change significantly suggesting the iso-structural phase, however, the values also increased a little with the increasing content of M^+^ ions. We proposed that the phase structures become more rigid with a high symmetry compared to the original Mg_2_Al_4_Si_5_O_18_ phase, so that the energy transfer possibility among Eu^2+^ decreases, which in turn decrease the possible non-radiative transition and lead to the increasing lifetime values in such a system.

**Figure 6:**
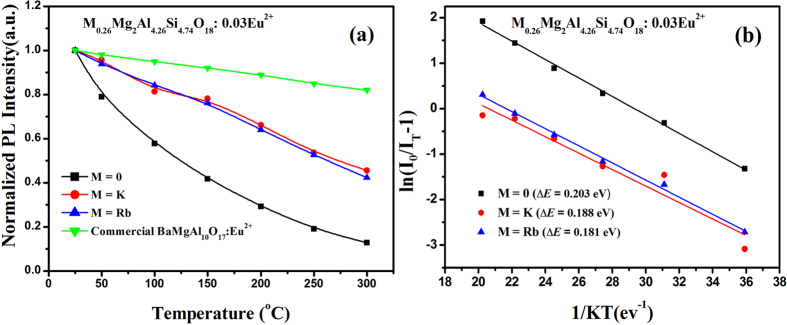
Temperature-dependent relative emission intensities (**a**) and the corresponding activation energy (*ΔE*) (**b**) of M_*x*_Mg_2_Al_4+*x*_Si_5−*x*_O_18_:0.03Eu^2+^ (M = K, Rb) phosphors.

Temperature dependence of PL spectra for M_*x*_Mg_2_Al_4+*x*_Si_5−*x*_O_18_:0.03Eu^2+^ (M = 0, 0.26 K and 0.26Rb) were studied in comparison to the commercial blue-emitting phosphor BaMgAl_10_O_17_:Eu^2+^ (BAM), and their variations are depicted in Fig. 6a. The relative emission intensities of all phosphor samples decrease with increasing temperature and the thermal degradation of samples is enhanced with the incorporation of K^+^ and Rb^+^ in the host. We observed the intensities’ decay of 41.6% for Mg_2_Al_4_Si_5_O_18_:0.03Eu^2+^, 78.1% for K_0.26_Mg_2_Al_4.26_Si_4.74_O_18_:0.03Eu^2+^, 76.2% for K_0.26_Mg_2_Al_4.26_Si_4.74_O_18_:0.03Eu^2+^ and 92.1% for BAM at 150 °C. Though the thermal stability of M_*x*_Mg_2_Al_4+*x*_Si_5−*x*_O_18_:0.03Eu^2+^ (M = 0, 0.26 K and 0.26Rb) is inferior to that of BAM, this phosphor has comparatively good temperature quenching effect and the thermal stability can be further enhanced via the optimization of composition and preparation experiment. Generally, the thermal quenching of emission intensity can be explained by the temperature dependence of the electron-phonon interactions in the luminescence center and thermally activated photo-ionization of lanthanide. These two mechanisms are strongly related to the crystal structure of host lattices and crystallinity of the phosphors, and are based on the observed thermal quenching rates^13^,^14^. In order to give a quantitative analysis of the thermally stable luminescence behaviors, the Arrhenius equation was employed to calculate the respective activation energy as follows^15^,^16^:


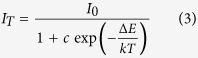


where *I*_0_ is the initial PL intensity of the phosphor at room temperature, *I*_*T*_ is the PL intensity at different temperatures, *c* is a constant, *ΔE* is the activation energy for thermal quenching, and *k* is Boltzmann constant (8.62 × 10^−5^ eV). According to the equation, the activation energy *ΔE* can be calculated from a plotting of ln[(*I*_*0*_*/I*)*−1*] against 1/*kT*, where a straight slope equals –*ΔE.* As shown in Fig. 6b, ΔE values were obtained to be 0.203 eV for Mg_2_Al_4_Si_5_O_18_:0.03Eu^2+^, 0.188 eV for K_0.26_Mg_2_Al_4.26_Si_4.74_O_18_:0.03Eu^2+^, and 0.181 eV for Rb_0.26_Mg_2_Al_4.26_Si_4.74_O_18_:0.03Eu^2+^, respectively, which are consistent with the evaluation of the thermal stability.

## Discussion

**Figure 7:**
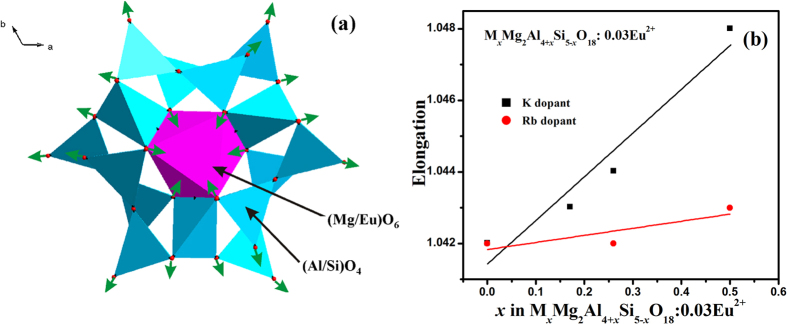
(**a**) The first and second coordination sphere of Mg/Eu ion. Small green arrows show approximate moving direction of O ions due to the enlargement of [(Al/Si)O_4_] tetrahedra. Bond length d(Mg/Eu–O) expected to be reduced in this case; (**b**) Relationship between elongation and *x* of M_*x*_Mg_2_Al_4+*x*_Si_5−*x*_O_18_:0.03Eu^2+^ phosphors.

It is well-known that red-shift behavior of Eu^2+^ emission is supposed to appear in M_*x*_Mg_2_Al_4+*x*_Si_5−*x*_O_18_:0.03Eu^2+^ with increasing M^+^ concentration. One explanation for this phenomenon is that addition of M^+^ dopants leads to the expansion of the tetrahedra [Al/SiO_4_] and then the values of *d*(Mg/Eu–O) decrease (Fig. 7a), so that red-shift in PL spectra can be observed. However, as can be seen in Fig. S3, cell volume increases with increasing *x*, and this causes increasing of *d*(Mg/Eu–O). The simultaneous increasing and decreasing of *d*(Mg/Eu–O) results in nonlinear bond length behavior for bond lengths of *d*(Mg/Eu–O) (Figure S4). This nonlinear behavior can lead to blue shift and after that to red shift with increasing *x*, but only red shift observed for these present phosphors. So, a new mechanism on red shift is ascribed to the distortion of [Mg/EuO_6_] octahedra due to the increasing concentration *x* of M^+^. Since all bond lengths *d*(Mg/Eu–O) in one crystal structure are the same, so we can calculate only elongation of octahedra by using PLATON program^17^. One can see from Fig. 7b that elongation increases with increasing of *x*. As far as the elongation is part of distortion, so increasing of the elongation leads to red shift of PL spectra. Hence, there are at least two factors which influence on PL spectra with increasing *x*: enlarging of [Al/SiO_4_] tetrahedra and elongation of [Mg/EuO_6_] octahedra. For studied compounds the elongation effect shows main effect on PL spectra resulting in red shift.

In addition, the internal quantum efficiency of three selected phosphors measured under 365 nm excitation at room temperature, and the measured QE values (internal QE) are 37.8%, 84.2% and 74.9%, respectively, for Mg_2_Al_4_Si_5_O_18_:0.03Eu^2+^, K_0.5_Mg_2_Al_4.5_Si_4.5_O_18_:0.03Eu^2+^, Rb_0.5_Mg_2_Al_4.5_Si_4.5_O_18_:0.03Eu^2+^, which are consistent with the variation of the PL emission intensities. As a reference, we have also measured the QE value of BAM, and the value is 48.3% under 365 nm excitation. These results indicated that the QE can be greatly enhanced by introducing M^+^ ions into the channels of Mg_2_Al_4_Si_5_O_18_:Eu^2+^, and these new solid solution phosphors can be potential in the practical use.

In summary, the new solid-solution phosphors M_*x*_Mg_2_Al_4+*x*_Si_5−*x*_O_18_:Eu^2+^ (M = K, Rb) were prepared by the sol-gel method. The continuous iso-structural phases have been determined based on Rietveld refinement. By gradually introducing M^+^ ions into the void channels, the emission intensities, thermal stability and QE were enhanced greatly compared to Mg_2_Al_4_Si_5_O_18_:0.03Eu^2+^. The emission peaks of M_*x*_Mg_2_Al_4+*x*_Si_5−*x*_O_18_:Eu^2+^ (M = K, Rb) phosphors performed a red-shift, which is mainly ascribed to that the elongation of [MgO_6_] octahedra increases with increasing of *x*. As such, this newly developed phosphors M_*x*_Mg_2_Al_4+*x*_Si_5−*x*_O_18_:Eu^2+^ (M = K, Rb) show great potential for use in high efficiency, thermally stable white LEDs.

## Methods

### Sample preparation

All the M_*x*_Mg_2_Al_4+*x*_Si_5−*x*_O_18_:0.03Eu^2+^ (M = K, Rb; 0 ≤ *x* ≤ 0.5) samples were synthesized by using the sol-gel method. Eu_2_O_3_ and Rb_2_O_3_ (99.995%, China Minls (Beijing) Research Institute, Beijing, China) were dissolved in HNO_3_ to obtain soluble Eu(NO_3_)_3_ and RbNO_3_ solutions. KNO_3_, Mg(NO_3_)_2_ and Al(NO_3_)_3_ were purchased from Sinopharm Chemical Reagent Co. Ltd., Shanghai, China, and the stoichiometric amounts of them were dissolved in ethanol under stirring. After this, the designed amounts of Si(OC_2_H_5_)_4_ (Sinopharm Chemical Reagent Co. Ltd., Shanghai, China) were added successfully in the above solution. The resultant mixtures continued to be stirred at 80 °C for 30 min, and then heated at 110 °C for 10 h in an oven until homogeneous gels were formed. After being dried, the gels were ground and treated at 900 °C for 10 h in the air, and then fully ground and sintered at 1330 °C for 4 h under a 10%H_2_-90%N_2_ gas mixture. Finally, they were furnace-cooled to room temperature, and ground again into powder for the following measurement.

### Structure and optical measurements

The powder X-ray diffraction (XRD) analysis were conducted on a D8 Advance diffractometer (Bruker Corporation, Germany) operating at 40 kV and 40 mA with Cu Kα radiation (λ = 0.15406 nm), and the scanning rate was fixed at 4^o^/min. The powder diffraction pattern for Rietveld analysis was collected with the same diffractometer. The step size of 2*θ* was 0.016°, and the counting time was 1 s per step. Rietveld refinement was performed by using TOPAS 4.2 software. High resolution transmission electron microscopic (HRTEM) images were characterized by a JEOL JEM-2010 microscope with an accelerated voltage of 200 kV. Room temperature excitation and emission spectra were measured on a fluorescence spectrophotometer (F-4600, HITACHI, Japan) with a photomultiplier tube operating at 400 V, and a 150 W Xe lamp used as the excitation lamp. The decay curves were recorded on an Edinburgh instrument (FLSP920) with a nF900 flash lamp was used as the excitation resource. The temperature-dependence luminescence properties were measured on the same F-4600 spectrophotometer, which was combined with a self-made heating attachment and a computer-controlled electric furnace (Tianjin Orient KOJI Co., Ltd, TAP-02). Quantum efficiency was measured using the integrating sphere on the FLSP920 fluorescence spectrophotometer (Edinburgh Instruments Ltd., UK).

## Additional Information

**How to cite this article**: Zhou, J. *et al.* New Insight into Phase Formation of M*x*Mg_2_Al_*4+x*_Si_5–*x*_O18:Eu^2+^ Solid Solution Phosphors and Its Luminescence Properties. *Sci. Rep.* **5**, 12149; doi: 10.1038/srep12149 (2015).

## Supplementary Material

Supplementary Information
