# Supplementary material for: New Insight into Phase Formation of MxMg2Al4+xSi5−xO18:Eu2+ Solid Solution Phosphors and Its Luminescence Properties
Source: Sci Rep. 2015 Jul 20;5:12149. doi: 10.1038/srep12149 (PMC4507260; doi:10.1038/srep12149)
Supplement: Supplementary Information [file srep12149-s1.pdf]

Supporting Information

New Insight into Phase Formation of $M_xMg_2Al_{4+x}Si_{5-x}O_{18}:Eu^{2+}$ Solid Solution Phosphors and Its Luminescence Properties

Jun Zhou[1], Zhiguo Xia[1,2*], Mingyue Chen[2], Maxim S. Molokeev[3,4], Quanlin Liu[2]

[1]*School of Materials Sciences and Technology, China University of Geosciences, Beijing 100083, China*

2School of Materials Sciences and Engineering, University of Science and Technology Beijing, Beijing 100083, China

3Laboratory of Crystal Physics, Kirensky Institute of Physics, SB RAS, Krasnoyarsk 660036, Russia

[4]*Department of Physics, Far Eastern State Transport University, Khabarovsk, 680021 Russia*

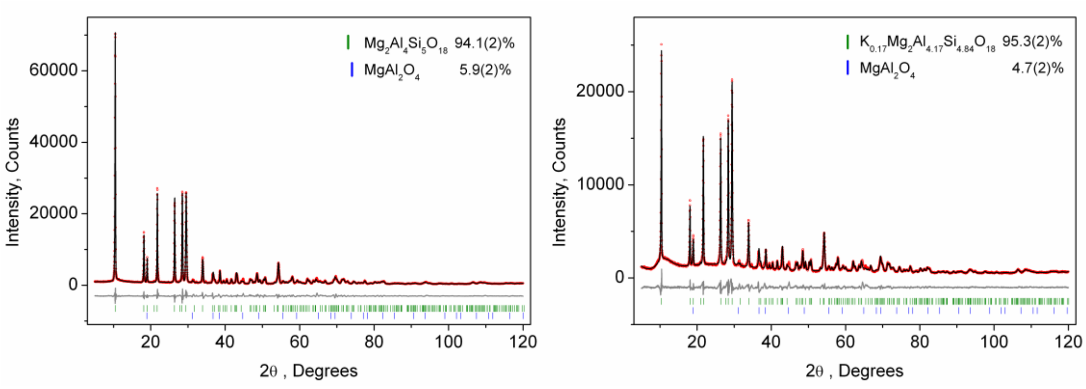

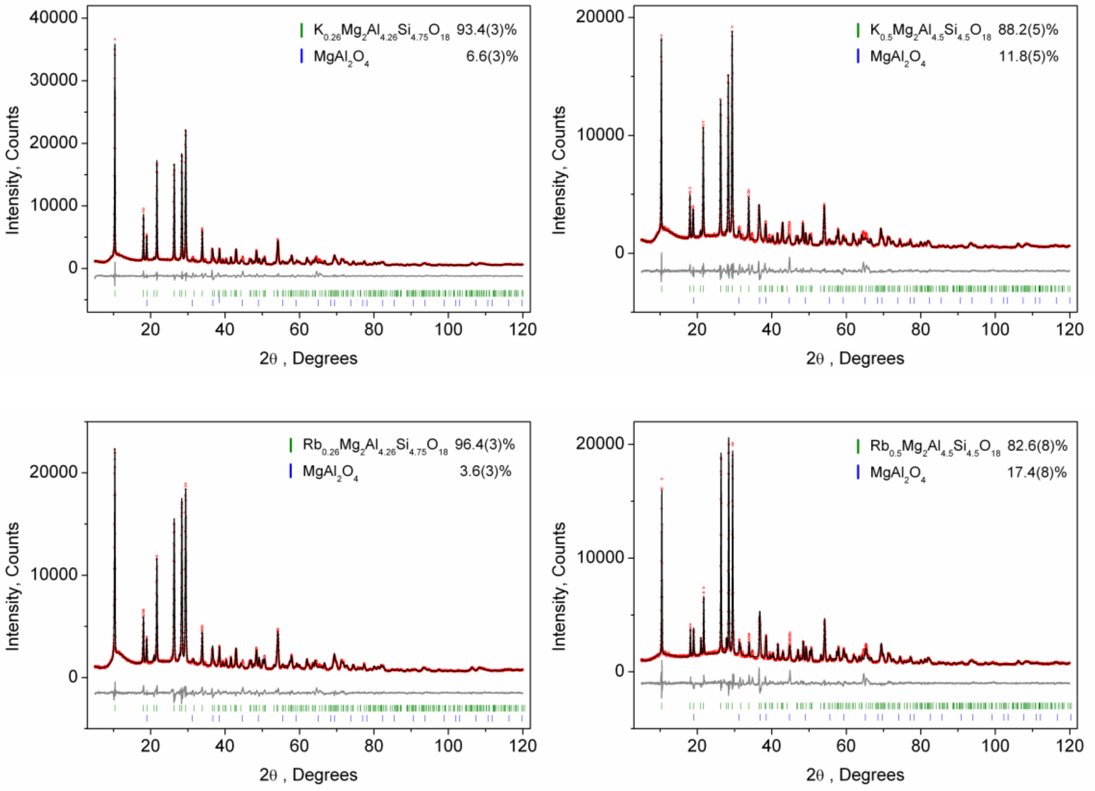

FIG. S1. Rietveld refinement of the powder XRD profile of $M_xMg_2Al_{4+x}Si_{5-x}O_{18}$ phosphors. Observed intensities (red circles), calculated patterns (black line), Bragg positions (blue mark), and the different plot (gray line) are shown for the Rietveld refinement.

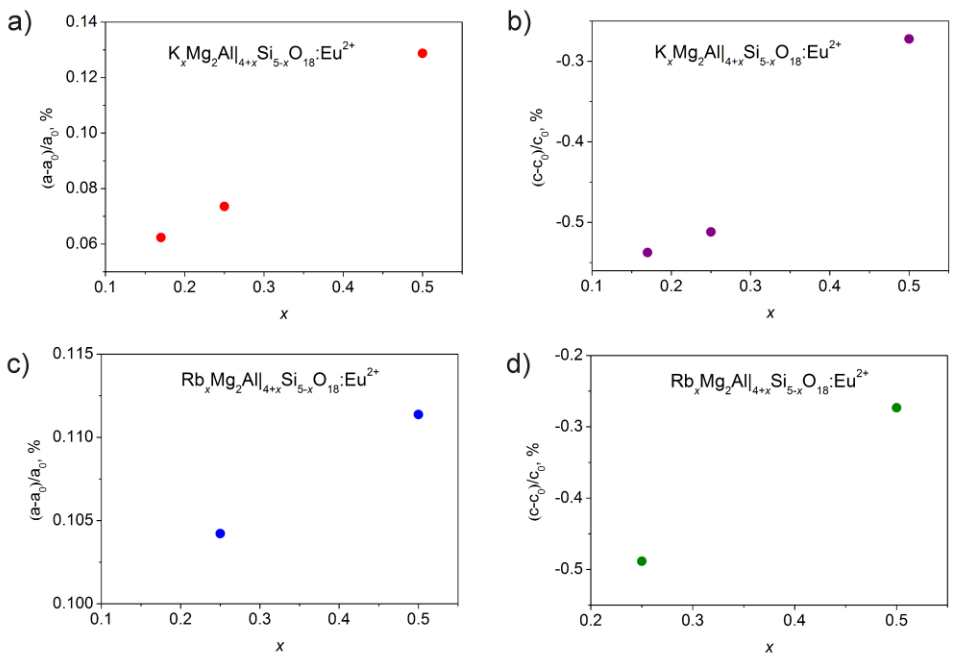

FIG. S2. Lattice variation of the $M_xMg_2Al_{4+x}Si_{5-x}O_{18}$ (M = K, Rb) phase compared to the original $Mg_2Al_4Si_5O_{18}$ phase: lattice mismatch of *a* (a) and *c* (b) values for $K_xMg_2Al_{4+x}Si_{5-x}O_{18}:Eu^{2+}$ series, and lattice mismatch of *a* (c) and *c* (d) values for $Rb_xMg_2Al_{4+x}Si_{5-x}O_{18}:Eu^{2+}$ series.

Table S1 Crystallographic data for $M_xMg_2Al_{4+x}Si_{5-x}O_{18}$ as determined from Rietveld refinement.

**Formula**	**$Mg_2Al_4Si_5O_{18}$**		**M=K**			**M=Rb**
x	0	0.17	0.26	0.5	0.26	0.5
**Space Group**	*P6/mcc*	*P6/mcc*	*P6/mcc*	*P6/mcc*	*P6/mcc*	*P6/mcc*
***a* (Å)**	9.7870(4)	9.7931(4)	9.7942(5)	9.7996(5)	9.7972(4)	9.7979(4)
***c* (Å)**	9.3374(5)	9.3389(5)	9.3413(5)	9.3638(5)	9.3435(5)	9.3637(5)
***V* ($Å^3$)**	774.56(6)	775.64(6)	776.03(7)	778.76(7)	776.69(7)	778.48(6)
**2θ-interval, °**	5-120	5-120	5-120	5-120	5-120	5-120
Z			2			
**R_{wp}, %**	9.99	7.35	7.64	8.44	6.53	7.71
**R_p, %**	7.28	5.37	5.21	5.76	4.88	5.30
**χ^2**	3.53	2.65	2.68	2.90	2.40	2.79

**$Mg_2Al_4Si_5O_{18}$ (*x*=0)**					
atom	*x*	*y*	*z*	B_{iso}	Occ.
Mg	1/3	2/3	1/4	1.07 (15)	1
Si1	1/2	1/2	1/4	1.05 (12)	5/9
Al1	1/2	1/2	1/4	1.05 (12)	4/9
Si2	0.3711 (2)	0.2677 (3)	0	0.92 (12)	5/9
Al2	0.3711 (2)	0.2677 (3)	0	0.92 (12)	4/9
O1	0.4833 (4)	0.3486 (4)	0.1455 (3)	1.17 (12)	1
O2	0.2239 (5)	0.3050 (4)	0	1.17 (12)	1

**$K_{0.17}Mg_2Al_{4.17}Si_{4.83}O_{18}$ (*x*=0.17)**					
atom	*x*	*y*	*z*	B_{iso}	Occ.
Mg	1/3	2/3	1/4	1.2 (2)	1
Si1	1/2	1/2	1/4	1.01 (17)	0.537

Al1	1/2	1/2	1/4	1.01 (17)	0.463
Si2	0.3711 (3)	0.2674 (3)	0	0.84 (17)	0.537
Al2	0.3711 (3)	0.2674 (3)	0	0.84 (17)	0.463
O1	0.4811 (4)	0.3467 (4)	0.1455 (3)	1.12 (17)	1
O2	0.2261 (6)	0.3078 (5)	0	1.12 (17)	1
K	0	0	0.356 (3)	5.0 (12)	0.085

**$K_{0.26}Mg_2Al_{4.26}Si_{4.74}O_{18}$ (*x*=0.26)**					
atom	*x*	*y*	*z*	B_{iso}	Occ.
Mg	1/3	2/3	1/4	0.71 (16)	1
Si1	1/2	1/2	1/4	0.77 (14)	0.527
Al1	1/2	1/2	1/4	0.77 (14)	0.473
Si2	0.3713 (3)	0.2675 (3)	0	0.81 (13)	0.527
Al2	0.3713 (3)	0.2675 (3)	0	0.81 (13)	0.473
O1	0.4820 (4)	0.3480 (4)	0.1456 (3)	0.93 (14)	1
O2	0.2257 (6)	0.3071 (5)	0	0.93 (14)	1
K	0	0	0.356 (2)	5.0 (8)	0.13

**$K_{0.5}Mg_2Al_{4.5}Si_{4.5}O_{18}$ (*x*=0.5)**					
atom	*x*	*y*	*z*	B_{iso}	Occ.
Mg	1/3	2/3	1/4	0.5 (2)	1
Si1	1/2	1/2	1/4	0.85 (19)	0.5
Al1	1/2	1/2	1/4	0.85 (19)	0.5
Si2	0.3713 (3)	0.2660 (4)	0	0.79 (18)	0.5
Al2	0.3713 (3)	0.2660 (4)	0	0.79 (18)	0.5
O1	0.4815 (5)	0.3470 (4)	0.1476 (4)	1.26 (19)	1
O2	0.2222 (7)	0.3037 (6)	0	1.26 (19)	1
K	0	0	0.3463 (15)	5.0 (6)	0.25

$Rb_{0.26}Mg_2Al_{4.26}Si_{4.74}O_{18}$ (*x*=0.26)					
atom	*x*	*y*	*z*	B_{iso}	Occ.
Mg	1/3	2/3	1/4	0.8 (2)	1
Si1	1/2	1/2	1/4	0.18 (18)	0.527
Al1	1/2	1/2	1/4	0.18 (18)	0.473
Si2	0.3707 (3)	0.2661 (3)	0	0.30 (18)	0.527
Al2	0.3707 (3)	0.2661 (3)	0	0.30 (18)	0.473
O1	0.4800 (4)	0.3462 (3)	0.1448 (3)	0.38 (18)	1
O2	0.2268 (5)	0.3064 (4)	0	0.38 (18)	1
Rb	0	0	0.3278 (9)	5.0 (4)	0.13

$Rb_{0.5}Mg_2Al_{4.5}Si_{4.5}O_{18}$ (*x*=0.5)					
atom	*x*	*y*	*z*	B_{iso}	Occ.
Mg	1/3	2/3	1/4	0.6 (2)	1
Si1	1/2	1/2	1/4	0.6 (2)	0.5
Al1	1/2	1/2	1/4	0.6 (2)	0.5
Si2	0.3694 (4)	0.2661 (4)	0	0.66 (19)	0.5
Al2	0.3694 (4)	0.2661 (4)	0	0.66 (19)	0.5
O1	0.4829 (4)	0.3462 (4)	0.1467 (5)	0.7 (2)	1
O2	0.2210 (8)	0.3011 (6)	0	0.7 (2)	1
Rb	0	0	0.2881 (14)	5.0 (4)	0.25

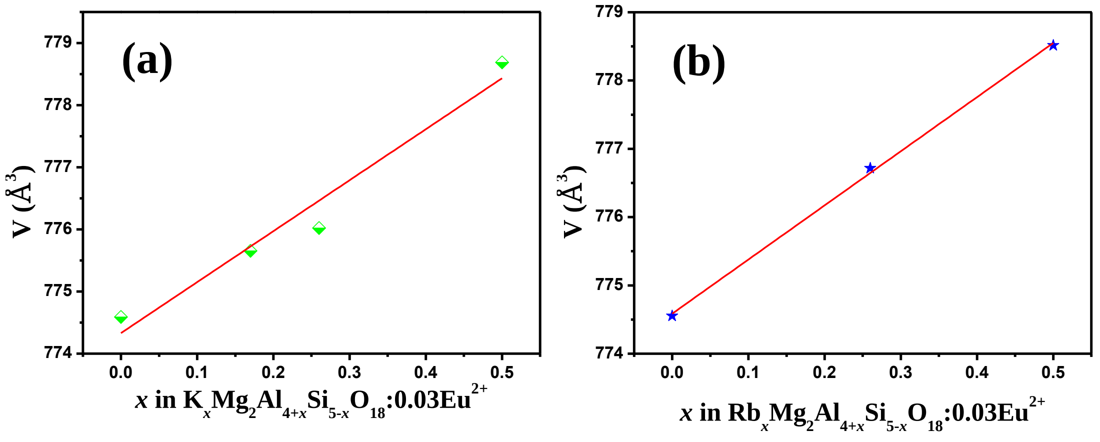

FIG. S3. Linear cell volume dependence per x in $K_xMg_2Al_{4+x}Si_{5-x}O_{18}$:0.03$Eu^{2+}$ (a) and $Rb_xMg_2Al_{4+x}Si_{5-x}O_{18}$:0.03$Eu^{2+}$ (b).

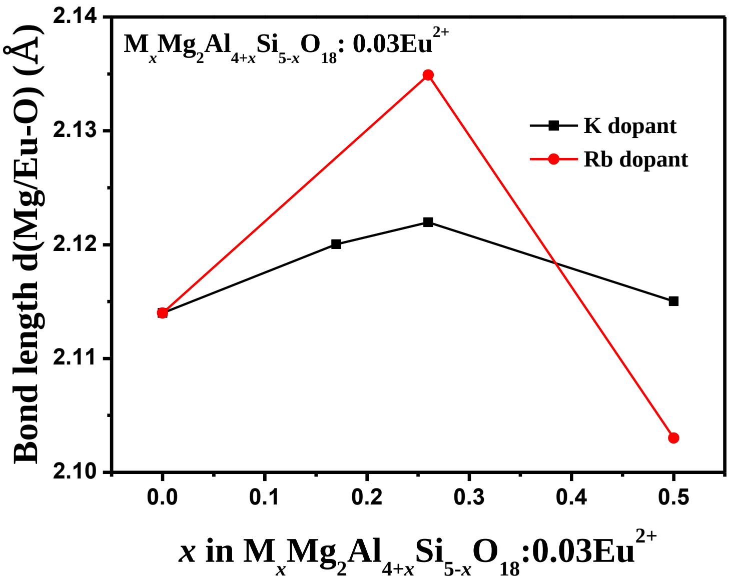

FIG. S4. Bond length d(Mg/Eu–O) per x in $M_xMg_2Al_{4+x}Si_{5-x}O_{18}$ compounds.
